# Graphene-Based Metamaterial Sensor for Pesticide Trace Detection

**DOI:** 10.3390/bios13050560

**Published:** 2023-05-19

**Authors:** Tingting Lang, Meiyu Xiao, Wenyang Cen

**Affiliations:** 1 School of Information and Electronic Engineering, Zhejiang University of Science and Technology, Hangzhou 310023, China; 2 Institute of Optoelectronic Technology, China Jiliang University, Hangzhou 310018, China; p20040854085@cjlu.edu.cn (M.X.); s20040803001@cjlu.edu.cn (W.C.)

**Keywords:** graphene, metamaterial, phosalone, terahertz

## Abstract

Organophosphate insecticides with broad spectrum and high efficiency make a great difference to agricultural production. The correct utilization and residue of pesticides have always been important issues of concern, and residual pesticides can accumulate and pass through the environment and food cycle, resulting in safety and health hazards to humans and animals. In particular, current detection methods are often characterized by complex operations or low sensitivity. Fortunately, using monolayer graphene as the sensing interface, the designed graphene-based metamaterial biosensor working in the 0–1 THz frequency range can achieve highly sensitive detection characterized by spectral amplitude changes. Meanwhile, the proposed biosensor has the advantages of easy operation, low cost, and quick detection. Taking phosalone as an example, its molecules can move the Fermi level of graphene with π–π stacking, and the lowest concentration of detection in this experiment is 0.01 μg/mL. This metamaterial biosensor has great potential in detecting trace pesticides, and its application in food hygiene and medicine can provide better detection services.

## 1. Introduction

Currently, there are many types of pesticides in the world, and the application of pesticides has brought considerable economic benefits to agriculture. However, the excessive utilization of pesticides has produced a serious influence on human health and the ecological environment. Taking organophosphate pesticides as an example, they are toxic to insects and mammals, and can easily damage their nervous systems [1]. In addition, organophosphate pesticides also inhibit the growth and reproduction of microorganisms in the soil, resulting in the dysfunction of ecosystems [2]. If pesticides are not used correctly and safely, there will be residual pesticides on the surface of crops that exceed the standard. Through the accumulation and spread in the environment and the food cycle, pesticide residues pose safety and health risks to humans and animals.

Traditionally, detection methods for pesticide residues include high-performance liquid chromatography, gas chromatography, and liquid chromatography–tandem mass spectrometry. Although these methods have high sensitivity, their expensive equipment, stringent experimental conditions, and long experimental time limit their applications [3,4,5,6,7,8,9]. Additionally, an enzyme-linked immunosorbent assay (ELISA) is also a highly sensitive method, but its use requires specific antibodies for detection. Given the thousands of pesticides and limited antibodies available, there is a need for new detection methods [7]. Since organophosphate pesticides can inhibit the activity of acetylcholinesterase (AChE), measuring AChE activity can serve as an indicator of the pesticide amount present, which is another way for detecting organophosphate pesticides [8,9]. However, strict operational conditions are required to keep enzyme activity intact. Consequently, achieving high-sensitivity, fast, and simple detection procedures remain challenges in detecting pesticide residues at trace levels.

Metamaterials, composed of sub-wavelength periodic unit cells, can produce abundant electromagnetic responses in the visible, terahertz, and infrared regions [10,11,12]. By artificially designing these metamaterials, they can modulate the phase and amplitude of incident light, making them suitable for use in filters, optical modulators, sensors, and absorbers, among other applications [13,14,15,16,17]. Many metamaterial sensors have been designed for substance detection [18,19]. Furthermore, metamaterials operating in the terahertz band provide a label-free and non-destructive detection method [20,21]. For example, carbon nanotubes can be used for sensing and detection [22,23], as well as various biochemical sensors [24].

Traditionally, metamaterial sensors consist of a dielectric layer and a metal layer, and they are sensitive to the refractive index of the top layer of the analyte. For example, a metamaterial sensor can measure 4-methylimidazole at concentrations from 0 to 20 mg/L, and the excited local electric fields effectively enhance the interaction between the incident terahertz light and the matter under testing [25]. By using a graphene-based metamaterial structure as a biosensor, the monolayer graphene can not only directly affect the excited local electric field but also move the Fermi level by π electrons of external molecules. Compared with traditional metal–dielectric metamaterial sensors, the introduction of the graphene layer can further enhance the interaction between the sensor and the analyte, enabling highly sensitive trace detection without any surface modification. During sensing, the movement of the Fermi level is equivalent to a special chemical doping of graphene, which impacts the resonance intensity, so amplitude changes can be achieved [26,27,28,29,30].

In this work, a graphene-based metamaterial biosensor working in the terahertz band is proposed. The sensor is composed of a polyimide (PI) film as the dielectric layer, an aluminum structure array as the metal layer, and monolayer graphene as the sensing interface. Simulation results indicate that the monolayer graphene, with varying Fermi levels, can modulate the entire electric field and produce the phenomenon of amplitude change. In addition, the adjustable amplitude range of the graphene-based metamaterial biosensor is almost independent of the structural parameters. Using phosalone as an example, the increasing concentration of the analyte results in a rise of the Fermi level of graphene, showing an increase in the transmission amplitude. The limit of detection (LOD) achieved in this experiment is 0.01 μg/mL. These findings demonstrate the potential of graphene-based metamaterial biosensors for trace detection of residual pesticides, and they may also be applied for detection devices in food hygiene and medical fields.

## 2. Structural Design

**Figure 1:**
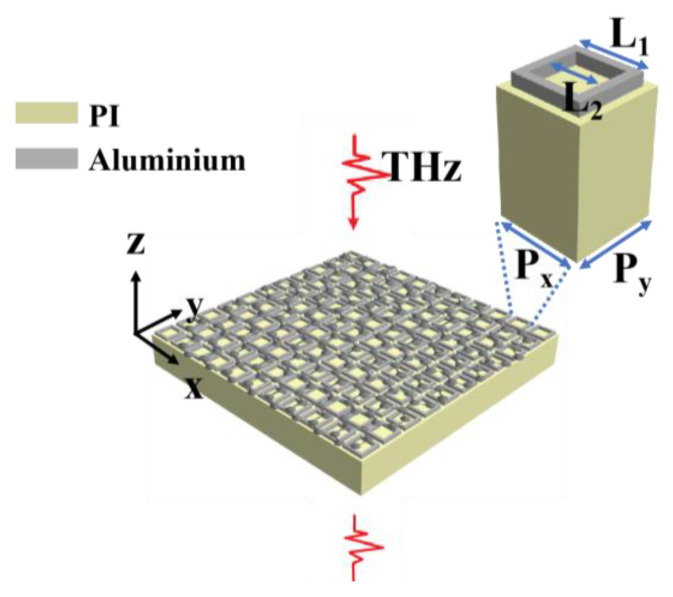
The model diagram of the graphene-based metamaterial biosensor.

The proposed model of the graphene-based metamaterial biosensor is depicted in Figure 1. It consists of three layers, a 50 μm PI layer, a 200 nm aluminum structure array layer, and monolayer graphene, which can be treated as an equivalent 2D impedance sheet. The unit cells have a period of P_x_ = P_y_ = 150 μm, and a square ring with an outer length of L_1_ = 122 μm and an inner length of L_2_ = 90 μm. The conductivity of the aluminum material is σ = 3.56 × 10^7^ Sm^−1^ [20]. Polyimide with permittivity ε = 2.98 − 0.165i (at 1 THz) was applied. In the THz band, the properties of various metals are similar. The price of aluminum is cheaper than gold and silver, and aluminum is less prone to oxidation than silver. The goal of this paper is to produce a pesticide sensor that can be mass-produced in the market and widely used in daily life, so aluminum is chosen as the most suitable metal material. There are four main reasons for using PI as the substrate: firstly, the loss of the THz wave in PI is small; secondly, the transmittance of the THz wave in PI can match that of silicon; thirdly, the PI substrate can overcome some defects of the traditional silicon substrate such as high hardness, inflexibility, and fragility; and fourthly, PI is cheap and suitable for mass production—the surface of PI is smooth and suitable for processing with traditional lithography technology. The Fermi level of the monolayer graphene was set at 0.1 eV. The simulation was conducted using CST Microwave Studio 2019, and the spectral curves in the terahertz band were calculated. Since the thickness of the monolayer graphene is theoretically 0.34 nm, which is much smaller than the wavelength in the terahertz band, its thickness can be ignored [31]. Prior to initiating the simulation, the beam propagation direction and the boundary conditions were defined. The terahertz waves were incident perpendicular to the surface of the metamaterial biosensor along the negative direction of the *z*-axis, with the electric field direction along the positive direction of the *x*-axis. The unit cell boundary condition was utilized both in the X and Y directions, and the open boundary was utilized in the Z direction to achieve a perfect absorption boundary.

## 3. Simulation Results

**Figure 2:**
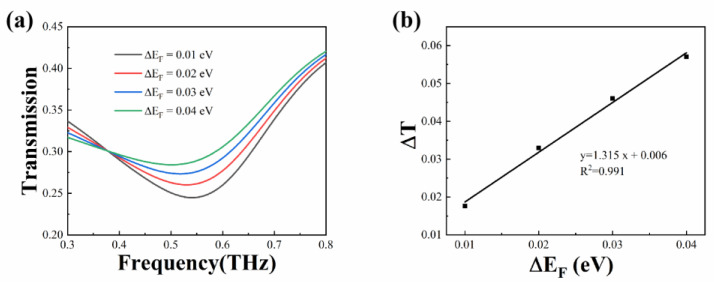
Simulation results of the graphene-based metamaterial biosensor with different Fermi level. (**a**) Transmission spectra when the Fermi level of the graphene ΔE_F_ ranges from 0.01 to 0.04 eV; (**b**) the linear fitting between ΔT and ΔE_F_.

The designed biosensor operates by detecting changes in the Fermi level of the graphene induced by the analyte, which in turn causes changes in the resonance strength [32,33]. In the simulation, the interaction between the molecules and the monolayer graphene is directly simulated by altering the Fermi level, as shown in Figure 2a. The initial Fermi level of the monolayer graphene is set at 0.1 eV. The transmission spectra with a Fermi level change of ΔE_F_ ranging from 0.01 eV to 0.04 eV are analyzed. As the Fermi level of graphene increases, the amplitude at the resonance frequency gradually rises, indicating that the resonance becomes weaker. The effective conductivity of the biosensor also increases as the Fermi level rises, which is the main reason for the weaker resonance. As shown in Figure 2b, within the range of ΔE_F_ from 0.01 eV to 0.04 eV, the change in transmission amplitude ΔT and ΔE_F_ exhibit a good linear relationship with a fitting degree of R^2^ = 0.99, which is beneficial for the following detection of substances.

**Figure 3:**
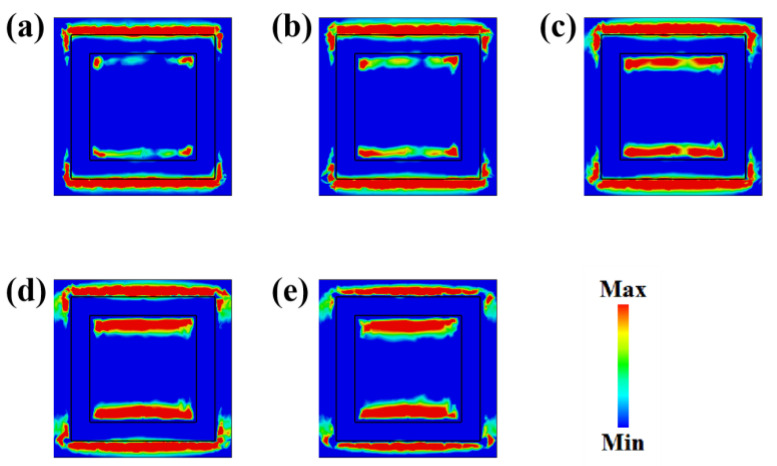
Simulation results of the surface electric fields when the monolayer graphene has different Fermi levels of (**a**) 0.10 eV; (**b**) 0.11 eV; (**c**) 0.12 eV; (**d**) 0.13 eV; and (**e**) 0.14 eV.

In addition, the surface electric fields on the top layer of the biosensor at the Fermi level of 0.10–0.14 eV are studied. As shown in Figure 3, it can be observed that as the Fermi level of graphene increases, the electric field at the outer edge of the square rings gradually weakens, while the electric field at the inner edge of the square rings gradually strengthens. The electric field at the outer edge is mainly caused by the neutralization of opposite charges from adjacent units, while the electric field at the inner edge is primarily caused by the neutralization of opposite charges within a unit cell. Based on the designed structural parameters, the opposite charges of adjacent unit cells are closer, so the electric field at the outer edge plays a dominant role. As the Fermi level of graphene increases, the weakening electric field at the outer edge leads to a weaker resonance, resulting in the phenomenon of increasing amplitude at the resonance frequency.

The structural parameters of the graphene-based metamaterial biosensor determine the intensity of the electromagnetic field that can be excited in the aluminum ring array layer. This, in turn, affects the range of transmission amplitude that can be controlled by the monolayer graphene. To investigate this effect, the outer and inner side lengths (L_1_ and L_2_, respectively) of the aluminum rings are selected as key parameters, and different spectra are analyzed. The change in transmission amplitude (ΔT) is set as the difference in amplitude when the Fermi energy level (ΔE_F_) changes. The larger the value of ΔT, the more sensitive the sensor is to changes in the Fermi level of graphene. Therefore, the influence of the structural parameters on the dynamic range of amplitude can show the change in sensitivity.

**Figure 4:**
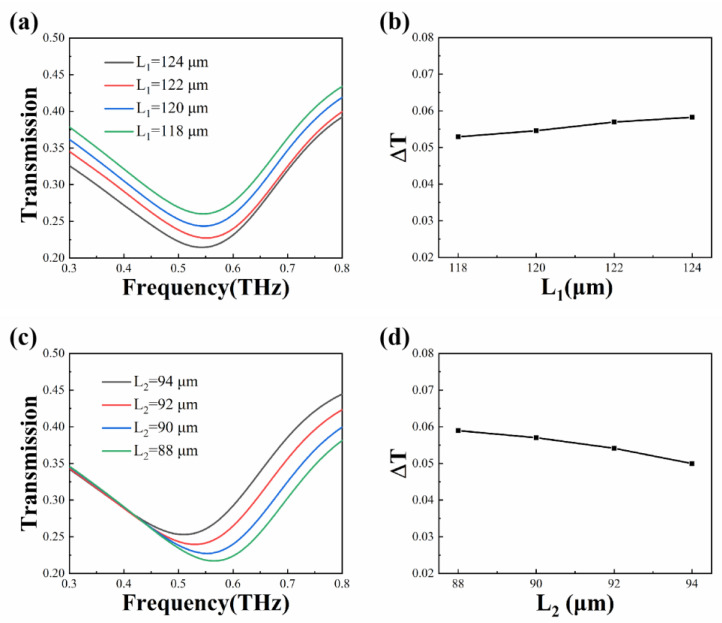
Simulated transmission spectra with different structural parameters. (**a**) Results for the graphene-based metamaterial biosensor with L_1_ ranging from 118 μm to 124 μm. (**b**) The trend of ΔT versus L_1_; (**c**) results for the graphene-based metamaterial biosensor with L_2_ ranging from 88 μm to 94 μm; (**d**) the trend of ΔT versus L_2_.

As shown in Figure 4a, with other parameters unchanged, the outer side length L_1_ is scanned in the range of 118 μm to 124 μm with a step size of 2 μm. As L_1_ increases, the transmission amplitude gradually decreases. As shown in Figure 4b, it can be seen that within the 6 μm range of change, the change in the transmission amplitude ΔT does not exceed 0.01. As shown in Figure 4c, the inner side length L_2_ is scanned in the range of 88 μm to 94 μm with the same step size of 2 μm, and the influence of L_2_ on the resonance frequency and transmission amplitude is more obvious. As shown in Figure 4d, it can be seen that within the 6 μm range of change, ΔT does not exceed 0.01. In summary, within a small range of variations, the tunable range of the graphene-based metamaterial biosensor can be slightly improved by increasing L_1_ and decreasing L_2_, which corresponds to the increasing line width of aluminum rings. However, it should be noted that the dynamic-tuning ΔT does not exceed 0.01 for a size variation of 6 μm. Therefore, it can be inferred that the tunable range of this biosensor is almost independent of the structural parameters of the metal layer, which indicates that the sensitivity of the biosensor is hardly affected.

## 4. Fabrication

**Figure 5:**
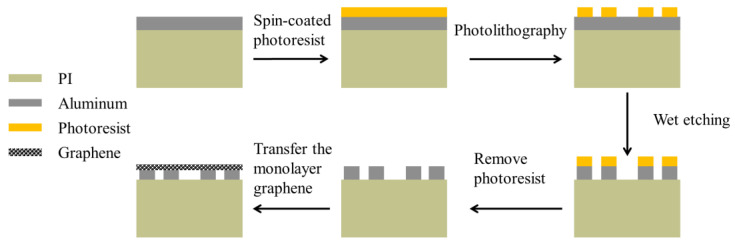
The fabrication process of the graphene-based metamaterial biosensor.

Furthermore, it is crucial to fabricate the biosensor based on the simulation design. This process mainly involves two steps: firstly, using photolithography and wet etching to fabricate the bare metamaterial composed of the dielectric and metal arrays, and secondly, transferring the monolayer graphene onto the surface of the metamaterial. The flow chart of the fabrication process is depicted in Figure 5.

**Figure 6:**
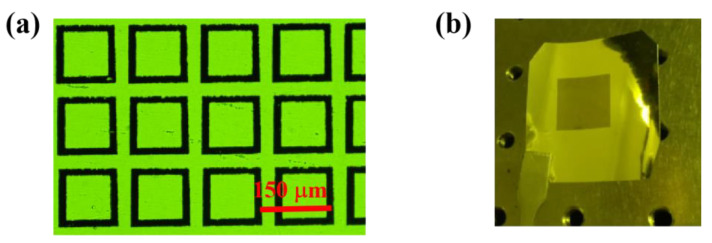
The fabricated graphene-based metamaterial biosensor. (**a**) The microscopic image of the biosensor. (**b**) The photograph of the biosensor.

As shown in Figure 6a, a transmission image of the bare metamaterial area was observed using an optical microscope, and slight scratches and irregularities in the boundaries of the aluminum rings can be observed. The scratches may have been caused by manual handling during film attachment, and the irregularities in the ring boundaries were due to the difficulty in controlling the consistency of the etching rate during the wet etching process. In the same amount of time, lines with a faster etching rate have smaller widths. Additionally, tiny impurities are likely to be unetched aluminum. As the monolayer graphene has a high visible light transmittance, there is no difference between the transmission images of the graphene-based metamaterial and the bare metamaterial. Figure 6b shows the entire graphene-based metamaterial biosensor. The area of the metamaterial biosensor is 12 × 12 mm^2^, and there are 80 × 80 units of the aluminum square rings. Finally, samples were measured with an average L_1_ length of 122 μm and an average L_2_ length of 90 μm.

**Figure 7:**
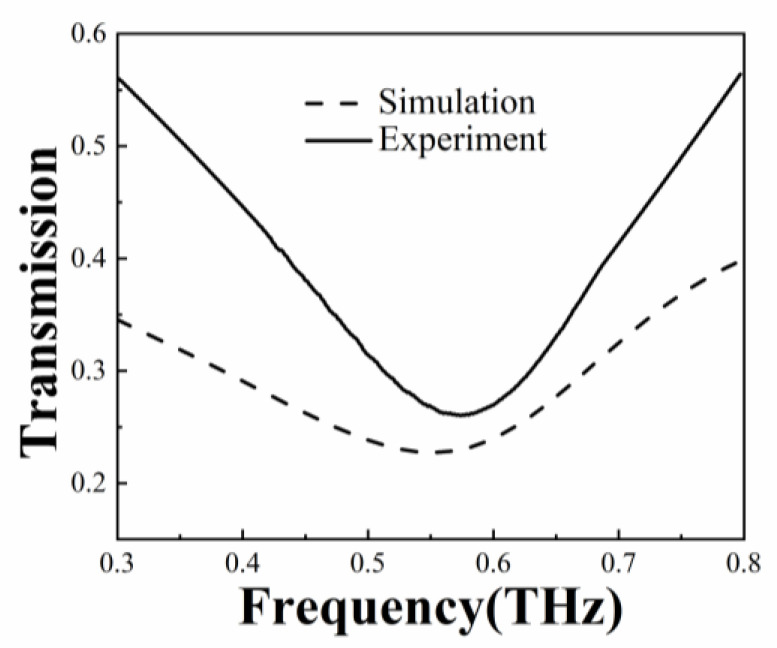
Comparison of simulated and experimental transmission spectra.

The monolayer graphene used in this experiment was purchased from Jicang-Nano Technology Co., Ltd. (Nanjing, China) with a size of 10 × 10 mm^2^, which determines the final sensing area. The transmission spectral measurements were conducted using a THz spectroscopy system (TeraScan 1550, Toptica Photonics AG, Gräfelfing, Germany) in the frequency range of 0.3 to 0.8 THz. As shown in Figure 7, the transmission spectra obtained from the simulation and experiment of the graphene-based metamaterial biosensor exhibit similar trends. The resonance frequency of 0.551 THz was obtained from the simulation and 0.574 THz from the experiment. Dimensional errors between the design and the actual values, and the irregular boundary of the aluminum rings, affect the localized electric field excitation, leading to a large difference in the full width at half maximum compared to the simulation.

## 5. Experimental Results

A standard solution of 10 μg/mL phosalone was purchased from Shanghai Aladdin Biochemical Technology Co., Ltd., Shanghai, China. This standard solution was diluted with acetone to prepare phosalone solutions with concentrations of 0.01 μg/mL, 0.05 μg/mL, 0.1 μg/mL, 1 μg/mL, and 2 μg/mL. These solutions were then applied on the surface of the biosensor for detection. Due to the fast evaporation rate of acetone, heating is not necessary during the drying process.

**Figure 8:**
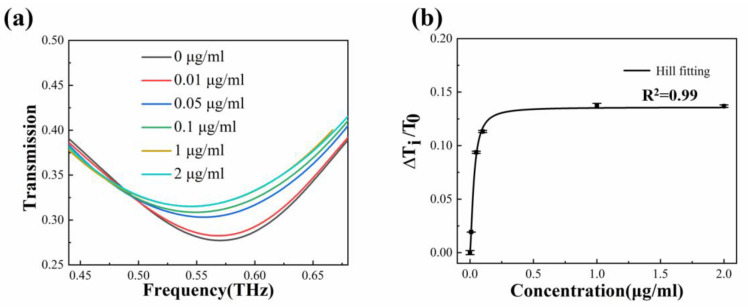
Detection of phosalone using the graphene-based metamaterial biosensor. (**a**) Experimental transmission spectra with different phosalone concentrations ranging from 0 to 2 μg/mL. (**b**) Fitting of experimental data according to the Hill model.

A pipette was used to drop 20 μL of phosalone solution onto the surface of the biosensor. As the solvent acetone quickly evaporates, the detection time is greatly reduced. Different spectral curves were obtained at different concentrations, as shown in Figure 8a. As the concentrations of the phosalone solutions increase, the transmission amplitude also gradually becomes larger, indicating an increasing Fermi level of the monolayer graphene. The relative change in transmission amplitude is defined as ΔT_i_/T_0_, where T_i_ and T_0_ represent the transmission amplitude at the resonance frequency in the i-th concentration and zero concentration of the analyte, respectively. After extracting the experimental data, ΔT_i_/T_0_ for phosalone solution concentrations of 0 μg/mL, 0.01 μg/mL, 0.05 μg/mL, 0.1 μg/mL, 1 μg/mL, and 2 μg/mL corresponds to 0%, 1.92%, 9.37%, 11.32%, 13.73%, and 13.69%, respectively.

The relative change in transmission amplitude is presented in Figure 8b. From the figure, it is evident that the relative change in transmission amplitude can be divided into two stages. Firstly, within the concentration range of 0–0.05 μg/mL, ΔT_i_/T_0_ increases rapidly and appears to maintain a linear relationship with the concentration of the solution. As concentrations further increase, ΔT_i_/T_0_ remains almost unchanged and exhibits a clear saturation effect. Therefore, the relative change in transmission amplitude caused by increasing solution concentrations is not a simple linear trend, which is common in biological sensing. To characterize the binding ability between the monolayer graphene interface and small biological molecules such as phosalone, the Hill model can be employed to fit the experimental data. The description of the Hill equation is as follows [34]:

(1) $$y=V_{\max}\frac{c^{n}}{k^{n}+c^{n}}$$

where V_max_ is the maximum ΔT_i_/T_0_ value at the saturation concentration, c is the concentration of the phosalone solution, n is the Hill coefficient, and k is the dissociation constant. The calculation can be performed using the commercial software Origin. The values of the parameters in Equation (1) were obtained by selecting the Hill function for fitting in the software Origin, and then the parameters were determined through the Levenberg–Marquardt iterative algorithm. The calculated V_max_ is 0.136, the Hill coefficient n is 1.558, the dissociation constant k is 0.032 μg/mL, and the goodness of fit is 0.999. The consistency between the experimental data and the fitting curve reflects the reliability and accuracy of the results.

The designed biosensor offers several advantages over traditional pesticide detection methods, such as low cost, ease of operation, and extremely short detection time. Moreover, it is more appropriate for biosensing at trace concentrations than traditional dielectric-metal metamaterials.

As shown in Table 1, compared with the same type of pesticide detector based on metamaterials, the graphene metamaterial biosensor has a low LOD and can be used to detect trace amounts of pesticides in daily production and life.

## 6. Conclusions

A graphene-based metamaterial sensor characterized by spectral amplitude changes is proposed. Taking the phosalone in organophosphorus insecticides as an example, the designed sensor has been simulated and experimentally verified. In the entire sensor structure, the PI film serves as the dielectric layer and the aluminum structure array acts as the metal layer, reducing cost. The monolayer graphene as the sensing interface further enhances the interaction between the surface and the analyte. The structural parameters of the metal layer do not significantly affect the adjustable amplitude range of graphene within a small variation range. Graphene can interact with molecules through π–π stacking, and graphene at different Fermi levels affects the electric field strength that can be excited by the entire metamaterial. Finally, the lowest concentration of detection LOD is 0.01 μg/mL, and the amplitude change in the concentration range of 0–2 μg/mL is in line with the Hill model. The graphene-based metamaterial biosensor has the advantages of a small size, high sensitivity, and label-free detection, making it a promising detection method for food safety industry applications.

## Figures and Tables

**Table 1:** Comparison of results with other sensors.

Type of Sensor	Pesticide Types	Band (THz)	LOD	Reference
Metamaterial absorber based on a split ring resonator structure	Indole-3-acetic acid and tricyclazole	0–3.5	10 ng/L	[35]
Loop-shaped absorber	Toxaphene	0.1–2	0.213 mg/L	[36]
All-dielectric grating metasurface	Chlorpyrifos	0.2–2.5	/	[37]
Graphene metamaterial sensor	Chlorpyrifos methyl	0.8–1.1	0.2 ng	[38]
All-dielectric broadband terahertz absorber	Chlorpyrifos	0.2–2	0.1 mg/L	[39]
Graphene-based metamaterial biosensor	Phosalone	0.3–0.8	0.01 mg/L	This work

## Data Availability

The data that support the findings of this study are available from the corresponding author upon reasonable request.
